# Cardiac deficiency of P21-activated kinase 1 promotes atrial arrhythmogenesis in mice following adrenergic challenge

**DOI:** 10.1098/rstb.2022.0168

**Published:** 2023-06-19

**Authors:** Eunjeong Jung, Rebecca Capel, Congshan Jiang, Elisa Venturi, Georgiana Neagu, Sarah Pearcey, Yafei Zhou, Yanmin Zhang, Ming Lei

**Affiliations:** ^1^ Department of Pharmacology, University of Oxford, Oxford OX1 3QT, UK; ^2^ National Regional Children's Medical Center (Northwest); Key Laboratory of Precision Medicine to Pediatric Diseases of Shaanxi Province; Xi'an Key Laboratory of Children's Health and Diseases, Shaanxi Institute for Pediatric Diseases; Xi'an Children's Hospital, Affiliated Children's Hospital of Xi'an Jiaotong University. No. 69, Xijuyuan Lane, Xi'an 710003, People's Republic of China; ^3^ Paediatric Intensive Care, Addenbrooke's Hospital, Cambridge CB2 1QY, UK; ^4^ Institute of Cardiovascular Sciences, Faculty of Medicine and Human Science, University of Manchester, Manchester, M13, 9GB UK; ^5^ Key Laboratory of Medical Electrophysiology of the Ministry of Education, Medical Electrophysiological Key Laboratory of Sichuan Province, Institute of Cardiovascular Research, Southwest Medical University, Luzhou, Sichuan, 646000, People's Republic of China

**Keywords:** p21-activated kinase 1, atrial fibrillation, atrial arrhythmia, isoprenaline, electrophysiology

## Abstract

P21-activated kinase 1 (Pak1) signalling plays a vital and overall protective role in the heart. However, the phenotypes of *Pak1* deficiency in the cardiac atria have not been well explored. In this study, *Pak1* cardiac-conditional knock-out (cKO) mice were studied under baseline and adrenergic challenge conditions. *Pak1* cKO mice show atrial arrhythmias including atrial fibrillation (AF) *in vivo*, detected during anaesthetized electrocardiography without evidence of interstitial fibrosis upon Masson's trichrome staining. Optical mapping of left atrial preparations from *Pak1* cKO mice revealed a higher incidence of Ca^2+^ and action potential alternans under isoprenaline challenge and differences in baseline action potential and calcium transient characteristics. Type-2 ryanodine receptor (RyR2) channels from *Pak1* cKO hearts had a higher open probability than those from wild-type. Reverse transcription-quantitative polymerase chain reaction and Western blotting indicated that pCamkII*δ* and RyR2 are highly phosphorylated at baseline in the atria of *Pak1* cKO mice, while the expression of *Slc8a2* and *Slc8a3* as a Na^+^–Ca^2+^ exchanger, controlling the influx of Ca^2+^ from outside of the cell and efflux of Na^+^ from the cytoplasm, are augmented. Chromatin immunoprecipitation study showed that pCreb1 interacts with *Slc8a2* and *Slc8a3*. Our study thus demonstrates that deficiency of *Pak1* promotes atrial arrhythmogenesis under adrenergic stress, probably through post-translational and transcriptional modifications of key molecules that are critical to Ca^2+^ homeostasis.

This article is part of the theme issue ‘The heartbeat: its molecular basis and physiological mechanisms’.

## 1. Introduction

An important aspect of atrial fibrillation (AF) pathophysiology is altered intracellular Ca^2+^ handling that contributes to both decreased contractile function and increased propensity to atrial arrhythmias, as well as structural remodelling (via activating hypertrophic signalling pathways) in the atria. Abnormal ‘phosphorylation states' of Ca^2+^-handling proteins occurs in several disease conditions including AF [1]. For example, changes in the phosphorylation level of the type-2 ryanodine receptor (RyR2) have been reported consistently in chronic AF. Phosphorylation of Ser2808 (Ser2809 depending on species) of RyR2 is higher in dogs with pacing-induced chronic AF and in chronic AF patients [2]. Protein kinase A (PKA)-overexpressing mice exhibit hyperphosphorylated RyR2 at Ser2808 (and hyperphosphorylated phospholamban at Ser16) and develop AF [3]. In human AF, increased CaMKII-dependent phosphorylation at Ser2815 of RyR2 leads to increased sarcolemmic reticulum (SR) Ca^2+^ leak, causing elevated cytosolic Ca^2+^ levels and providing a potential arrhythmogenic substrate. Goats with sustained AF show enhanced CaMKII autophosphorylation and thus increased activity of CaMKII along with higher CaMKII-dependent RyR2 phosphorylation at Ser2815 [4]. On the other hand, genetic inhibition of CaMKII phosphorylation of RyR2 in *RyR2^S2814A^* knockin mice reduced AF inducibility in a vagotonic AF model [5]. These data indicate a critical role of abnormal ‘phosphorylation states’ of Ca^2+^-handling proteins in AF. Understanding the controlling mechanisms of phosphorylation/dephosphorylation of Ca^2+^-handling proteins in atrial myocytes may thus help identifying new targets for developing novel mechanism-based therapeutic approaches for AF.

Our work and others’ over the past decade have led to the identification, in ventricular and pacemaker tissues/cells [6–13], of new roles of multifunctional enzyme P21-activated kinase-1 (Pak1) in the heart, in particular its role in regulating ion channel and Ca^2+^ handling protein function/activity through PP2A and other downstream effectors [6–13]. Pak1 is a key member of a highly conserved family of serine-threonine protein kinases (Pak family) regulated by Ras-related small G proteins, Cdc42/ Rac1.

In the cardiac ventricle, *Pak1* is required to maintain calcium homeostasis and electrophysiological stability via transcriptionally regulating SR Ca^2+^-ATPase 2a (SERCA2a). Cardiac deficiency of *Pak1* was thus shown to contribute to tachyarrhythmia susceptibility in the mouse intact ventricle and primary isolated myocytes following isoprenaline (ISO) challenge [14].

Pak1 function in atrial tissue/myocytes has less been explored. Work in recent years has shed new light on the unique calcium signalling in atrial myocytes. At an ultrastructural level, although atrial and ventricular myocytes share many similarities, there are several key structural differences, in particular the lack of transverse tubules (T-tubules) in atrial myocytes, which makes atrial myocytes display vastly different calcium patterns in response to depolarization than ventricular myocytes. The lack of T- tubules in atrial myocytes means that depolarization provokes calcium signals that largely originate around the periphery of the cells. Such differences in Ca^2+^ homeostasis between atrial and ventricular cells may be significant in determining the increased prevalence of arrhythmias in the atria [15]. An additional important difference between atrial and ventricular muscle concerns the resting activity of adenylyl cyclase: in atrial myocytes there is significant turnover in cAMP-dependent pathways even in the absence of β-adrenocpetor stimulation [16,17]. As a consequence, it is predicted that the roles of phosphatase activation by Pak1 will be enhanced in atrial tissue. One recent study by DeSantiago *et al*. [18] demonstrated that reduced Pak1 activity increases the inducibility of atrial arrhythmia *in vivo* and *in vitro*. On the cellular level, Pak1^−/−^ atrial myocytes exhibit increased basal and AngII (1 µM)-induced reactive oxygen species (ROS) production, sensitivity to the NOX2 inhibitors and enhanced membrane translocation of Rac1 (part of the multi-molecular NOX2 complex). They suggest these experiments support that Pak1 stimulation can attenuate Na^+^-Ca^2+^ exchanger (NCX)-dependent Ca^2+^ overload and prevent triggered arrhythmic activity by suppressing NOX2-dependent ROS production.

This study is designed to determine whether *Pak1* deficiency in cardiac atrial myocytes of mice could bring potential atrial dysfunction including AF via abnormal calcium handling and dysregulated signalling pathways. Thus, the present study uses a mouse model with conditional, cardiomyocyte-specific deletion of *Pak1* (*Pak1* cardiac-conditional knock-out (cKO)), firstly to clarify the role of *Pak1* signalling in regulating electrical function and Ca^2+^ handling in the atria under baseline and β-adrenoceptor stimulation, and secondly to investigate the implicated signalling pathways and key molecules leading to abnormal Ca^2+^ handling and SR function in *Pak1* cKO atria.

## 2. Material and methods

### (a) Experimental animals

*Pak1* cKO was achieved with Pak1^fl/fl^; α-MHC-Cre^+^ animal breeding as described in our previous work [13], and carried out after ethical approval under project licence PP8557407. All animal experiments were performed on adult mice in accordance with the United Kingdom Animals (Scientific Procedures) Act 1986, European Parliament Directive 2010/63/EU and were approved by the University of Oxford Pharmacology ethical committee (PPL: PP8557407) or Manchester University Research Ethics Committee (PPL: 40/3406) in conformity with the national guidelines under which the institution operates. *Pak1*^fl/fl^ was homozygous with the exon 3 of *Pak1* gene flanked with 2 loxp sites. Cre with α-myosin heavy chain (α-MHC) promoter was used for cardiac myocyte-specific Cre expression, while *Pak1*^fl/fl^; α-MHC-Cre^−^ were used as control mice. The breeding, genotyping and functional validation of both *Pak1* cKO and *Pak1*^fl/fl^ control mice were described in our previous work [13]. *Pak1* cKO and *Pak1*^fl/fl^ littermates used in this study were maintained in a pathogen-free facility at the University of Oxford and/or the University of Manchester. All mice were kept in individually ventilated cages at room temperature in a specific pathogen free animal facility, had free access to sterile rodent chow and water and 12 h light/dark cycles. The use of mice was shared between members of the laboratory, in which cardiac and other tissues were taken from the same mouse wherever possible to comply with the ‘three Rs’ principles. Only male mice were used for our experiments. All chemical agents were purchased from Sigma-Aldrich (Poole, UK) except where indicated. Age and gender matched four months old *Pak1* cKO and *Pak1*^fl/fl^ littermate control mice were used. *In* vivo ISO treatment was performed via intraperitoneal (i.p.) injection.

### (b) Surface electrocardiography

Surface electrocardiography (ECG) and *in vivo* electrophysiological studies were performed in *Pak1* cKO mice and age-matched controls (four months old). Surface ECG was recorded for 10 min under 2.5% isoflurane inhalation. Mice were placed on a 37°C heating pad with body temperature monitoring for three-lead ECG measurements using subcutaneous needle electrodes employing a PowerLab 26T system (AD Instruments, Hastings, UK). The digital recordings (16 bit, 2 kHz channel^−1^) were analysed using the Chart v6.0 program (AD Instruments, Oxfordshire, UK) giving signal-averaged ECGs and ECG parameters in which corrected QT was given as QTc = QT/(RR/100)^1/2^ in line with previous reports [19].

### (c) *In vivo* electrophysiology study

A series of *in vivo* experiments applied programmed stimulation procedures. This used an ultraminiature octapolar 1.1F electrophysiological catheter (EPR-800, Millar Instrument, Inc., Houston, TX, USA) inserted via the jugular vein and placed into the right atrium. The stimulation protocols were performed after 10 min of stable simultaneous recordings of the baseline surface ECG.

Pacing protocols including (i) sinus node recovery time (SNRT): deliver atrial pace at drive cycle lengths of 100 ms for 30 s. Three runs with 10 s intervals; (ii) atrioventricular (AV) node conduction: deliver atrial burst pacing starting at 150 ms and decremented by 5 ms every train and down to AV Wenckebach cycle length. Continue delivering atrial burst pacing and decrement by 5 ms down to 2 : 1 conduction; and (iii) atrial fibrillation threshold (AFT): three trains of 20 pulses at 20 ms cycle length, with 2 s interval between trains, starting from twice threshold and incrementally increased by 1 mA until induction of AF.

### (d) Histological analysis of atrial fibrosis

After *in vivo* physiological experiments, mice were sacrificed by cervical dislocation under Schedule 1 (UK Animals (Scientific Procedures) Act 1986). Hearts were washed in saline twice before weighing. Hearts were then fixed in 4% buffered formaldehyde for 48 h. After paraffin embedding and serial sectioning, sections (5 µm thickness) were stained with Masson's trichrome for interstitial fibrosis. Images were taken at 20× magnification with a three-dimensional Histec Pannoramic slide scanner. The Pannaoramic Viewer software was used with the 20× zoom setting and a snapshot was taken. For each section, non-overlapping photomicrographs were obtained from the entire left atrium. Representative images from all four animals from both groups were shown.

### (e) Optical mapping

Dual-dye optical mapping using voltage and Ca^2+^ sensitive dyes was used to explore changes in action potential duration (APD), refractory period, Ca^2+^ transients, onset of Ca^2+^ and voltage alternans and arrhythmias under control conditions and acute adrenergic stress in *Pak1* cKO and *Pak1*^fl/fl^ control mice. The voltage sensitive dye RH237 and the calcium indicator dye Rhod-2 AM, which have a shared excitation wavelength but different emission spectra were used for optical mapping. RH237 and Rhod-2 mixed with Pluronic F-127 were loaded by Langendorff perfusion by slow injection into a side-port and recordings were carried out in the presence of contraction uncoupler Blebbistatin at a concentration of 10 µM [20]. The high speed (1 kHz) dual-dye optical mapping system consists of a series of lenses focusing emitted light into an OptoSplit beam-splitter (Cairn Research Ltd, UK), to separate light emitted by each dye based on wavelength, and a camera (Photometrics Evolve 128) with a spatial resolution of 74 × 74 µM per pixel and a temporal resolution of 1000 frames s^−1^. A 10 nM dose of ISO was chosen for cardiac slices as it has previously been used in whole-heart work to produce a robust β-adrenergic response without consistently inducing arrhythmias in healthy wild-type hearts [21]. The atria were removed from the whole heart and pinned into a Sylgard-coated bath. Any overlying tissue was dissected away, the left atrium was positioned in the field of view and stimulated on the epicardial surface using point-stimulation with an Ag electrode at twice the threshold voltage. During the S1S2 protocol, used to determine refractory period, the tissue was paced with trains of eight stimuli of an 8 Hz frequency (the S1) interrupted by a ninth stimulus (the S2) which started at 125 ms but gradually shortened by 2.5 ms with every cycle applied. During the dynamic stimulation protocol, the tissue was stimulated continuously, starting at a cycle interval of 135 ms and the interval gradually decreasing by 5 ms every 20 impulses. The dynamic protocol was used to assess action potational (AP) and calcium transient characteristics, and susceptibility to the development of alternans. The threshold for amplitude alternans was set at an amplitude alternans ratio (AAR) of 0.15, where AAR = 1 − AL/AH (AH and AL represent the lower and higher transient amplitude) over a minimum of six consecutive beats. As a result, an average of three consecutive AARs above 0.15 indicated alternans occurrence.

### (f) RyR2 channels recording

#### (i) Isolation of mixed membrane from mouse cardiac tissue

Isolated mixed membrane vesicles were prepared from a mixture of male and female hearts from *Pak1* cKO or wild-type (WT) using methods described previously [22]. Mice were 10–13 weeks old. Briefly, mouse cardiac tissue was dissected and snap frozen. Frozen tissue was finely homogenized in a buffer containing 300 mM sucrose, 20 mM K^+^ piperazine-N,N′-bis(2-ethanesulfonic acid), 5 mM sodium fluoride (NaF), 2.5 mM dithiothreitol, 1 mM phenylmethylsulfonyl fluoride, supplemented with a protease inhibitor cocktail, pH 7.2. The tissue homogenate was centrifuged at 6000*g* for 20 min at 4°C. The supernatant obtained was centrifuged at 120 000*g* for 1 h at 4°C to pellet the membrane fraction. This was resuspended in 400 mM sucrose, 5 mM Tris/HEPES, 5 mM NaF at pH 7.2. The preparation was snap frozen and stored at −80°C.

#### (ii) Single-channel recordings

RyR2 channels were incorporated into planar phospholipid bilayers as previously described [23] in a 1 : 1 mixture of phosphatidylethanolamine : phosphatidylserine (Avanti Lipids). Current fluctuations through RyR2 channels were recorded under voltage-clamp conditions with 250 mM HEPES, 80 mM Tris, 2 µM free Ca^2+^, pH 7.2, on the cytoplasmic side and 250 mM glutamic acid, 10 mM HEPES, pH to 7.2 with Ca(OH)_2_ (free [Ca^2+^] approximately 50 mM) on the trans (luminal) side of the bilayer at 21°C. The luminal chamber was voltage-clamped at ground. The free [Ca^2+^] and pH of the solutions were measured using a Ca^2+^ electrode (Orion 93-20, Thermo Fisher Scientific, UK) and a Ross-type pH electrode (Orion 81-55, Thermo Fisher Scientific, UK) as previously described [23].

#### (iii) Single-channel analysis

Single-channel recordings were digitized at 20 kHz and low-pass filtered at 800 Hz. Open probability (Po) was determined over 3 min of continuous recording using 50% threshold analysis (REF COLQ + SIG 1995) [24] in Clampfit (Molecular Devices, USA). The Po values indicated above the representative traces shown in the figures were calculated from the full 3 min recording for that channel. Where greater than one channel incorporated into the bilayer, Po is reported as average Po (total Po/no. of channels).

### (g) Reverse transcription-quantitative polymerase chain reaction

Messenger RNA (mRNA) expression levels of *RyR2, Atp2a2 (Serca2a), Cacna1c (Ltcc)*, AP1 components including *cFos* and *cJun* genes, different *Slc8a* isotypes including *Slc8a1-3* were detected in atria of *Pak1* cKO and *Pak1*^fl/fl^ control mice using reverse transcription-quantitative polymerase chain reaction (RT-qPCR). Total RNA from cells were isolated with TRIzol (Invitrogen Co., Ltd.) and reverse transcribed into in a total volume of 10 *μ*l (500 ng) using the ReverTra Ace qPCR RT Master Mix (Toyobo Life Science) following previous studies [25]. The primer information is described in the electronic supplementary material, table S1 from the electronic supplementary material, data file. The expression levels of genes were normalized to those of GAPDH and calculated using the 2^−ΔΔCt^ method.

### (h) Western blotting

To investigate whether Atria of *Pak1* deficient mice show the increased phosphorylation of CamkII and RyR2, we treated *Pak1*^fl/fl^ and *Pak1* cKO mice with ISO (i.p. 10 mg kg^−1^), extracted atria following ISO treatment, and observed phosphorylation of CamkII*δ* (T287), RyR2 (S2814) and RyR2 (S2808). To determine the protein expression levels, Western blotting experiments were performed as described in our previous study [26]. Heart tissues were lysed with ice-cold RIPA buffer (Thermo Fisher Scientific, Inc.) supplemented with protease and phosphatase inhibitor cocktail (Thermo Fisher Scientific, Inc.). Twenty micrograms total protein for each lane was resolved by 5% stacking gel and 10% or 6% separation gel, and transferred to a 0.45 µm polyvinylidene fluoride membrane. The membrane was blocked for 2 h with 5% non-fat dry milk in Tris-buffered saline with tween buffer, and was incubated with the primary antibody at 4°C overnight. The primary antibodies included anti-phosphorylated-CamkII (T287) (ab182647, Abcam, UK), anti-total CamkII (sc-100362, Santa Cruz biotech, USA), anti-phosphorylated-RyR2 (S2814) (A010-31, Badrilla Ltd., UK), anti-phosphorylated-RyR2 (S2808) (A010-30AP, Badrilla Ltd., UK) and anti-total-RyR2 (ab2868, Abcam, UK). The membrane was incubated with the horse anti-mouse IgG horseradish peroxidase (HRP) (no. 7076, Cell Signaling, USA) and goat anti-rabbit IgG HRP (no. 7074, Cell Signaling, USA) secondary antibody for 1 h at room temperature. Images were captured and immunoreactive bands were quantified using Quantity One software v4.6.6 (Bio-Rad Laboratories, Inc.).

### (i) Chromatin immunoprecipitation assay

Chromatin immunoprecipitation assay (ChIP) was performed to identify the potential interactions of *Slc8a2* and *Slc8a3* directly bind with pCreb1 (cAMP element response binding protein, no. 9198, Cell Signalling, USA). The antibody of pCreb1(S133) was reacted at 1 : 50 dilution. The fixation solution, cell lysis buffer, and nuclear lysis buffer, and other buffers were prepared according to Wiehle’ experiment [27]. Firstly, fixation solution was added to cardiomyocytes, and the cell pellet resuspended with ice-cold cell lysis buffer to perform cross-linking and chromatin shearing. Cells were collected and 0.4 ml of glass beads were added to the cell suspension to precede the immunoprecipitation and reversal of cross-links. Following these stages, traditional PCR is the preferred method for analysing known target regions. 1 kb DNA ladder (no. SM1163, Thermo Fisher Scientific, UK) was used for a DNA marker.

### (j) Statistical analysis

Data are displayed as mean ± s.e.m. One-way ANOVA method followed with Tukey-Kramer *post-hoc* test and two-way ANOVA method with Sidak *post-hoc* correction was chosen for comparison among multiple groups and Student's *t*-test were used to statistically compare the data between two groups. Fisher's exact test was used for categorical data. *p* < 0.05 was considered as statistically significant.

## 3. Results

### (a) *Pak1* deficiency increases atrial tachyarrhythmic susceptibility in living mice

**Figure 1.**
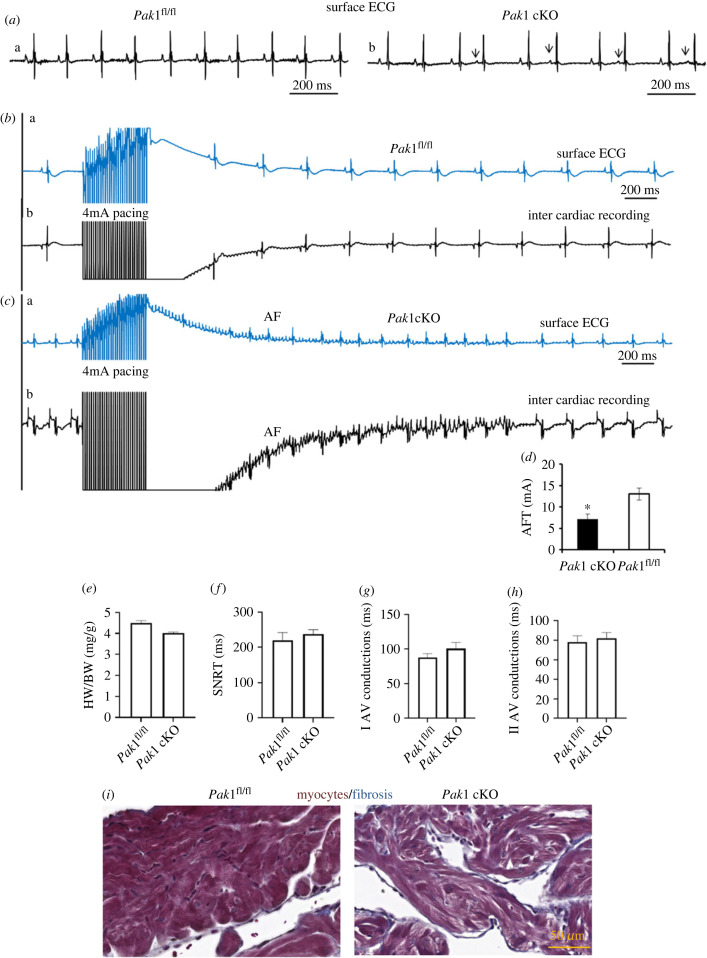
The *in vivo* electrophysiological recordings and left atria histology with Masson's trichrome staining in *Pak1* conditional knock out and control mice. (*a*) Surface ECG obtained from a *Pak1*^fl/fl^ control, a, and *Pak1* cKO, b, mouse. An episode of spontaneous atrial bigeminy with varied RR interval. Arrows indicate early onset of P waves, suggesting wandering atrial ectopic foci. (*b,c*) Simultaneous surface ECG and inter cardiac recording of P*ak1* cKO and *Pak1*^fl/fl^ control mice. Traces a (Ba and Ca) and b (Bb and Cb) refer to surface ECG lead I and simultaneous inter cardiac recording under AFT pacing protocol of 4 mA as pacing current. Representative imaging are shown. (*d*) Atrial fibrillation threshold (AFT) from *Pak1* cKO and *Pak1*^fl/fl^ control mice. (*e*) Heart weight versus body weight (HW/BW, mg g^−1^). (*f*) Sinus node recovery time (SNRT)(ms). (*g*) Type I AV conduction (ms). (*h*) Type II AV conduction (ms). *n* = 6 mice per group in (*a–d*), *n* = 3 to 4 mice per group in (*e–h*), bars represent mean ± s.e.m. (*i*) Masson's trichrome staining. Left column: *Pak1*^fl/fl^ control, right column: *Pak1* cKO. One representative image of *Pak1*^fl/fl^ control and *Pak1* cKO mice from all four analysed animals in both groups is shown. Scale bar: 50 µm.

The *in vivo* electrophysiological assays including simultaneous surface ECG and intercardiac recordings were performed in *Pak1* cKO and *Pak1*^fl/fl^ mice. The results showed that all ECG parameters (R-R, P-R, QRS, QT intervals) are indistinguishable between *Pak1* cKO and *Pak1*^fl/fl^ mice under baseline and adrenergic challenge (via i.p. injection of ISO at 2 mg kg^−1^) (table 1). Without treatment *Pak1* cKO mice demonstrated significantly high incidences of atrial arrhythmias than *Pak1*^fl/fl^ control mice (*p* < 0.001), but not in *Pak1*^fl/fl^ control mice (0 out of 43; figure 1*a*a), while atrial arrhythmias including atrial bigeminy, multi atrial ectopic beats, and atrial tachycardia (1 out of 28) episodes were detected in *Pak1* cKO mice (1, 1, and 5 out of 28 respectively) from resting surface ECG (figure 1*a*b). With ISO treatment, baseline ECG did not detect atrial arrhythmia except heart rate significantly increased in both *Pak1* cKO and *Pak1*^fl/fl^ control mice (*n* = 3 of each). Data from both the surface ECG and the intercardiac recordings showed that AF was not induced by right atrium AFT pacing protocol of 4 mA as pacing current in the hearts of controls (*n* = 6) (figure 1*b*a,b). However, AF was induced under AFT pacing protocol of 4 mA as pacing current in hearts of *Pak1* cKO mice (3 out of 6) (figure 1*c*a,b). The AFT of *Pak1* cKO mice was significantly lower (*p* < 0.05) (*n* = 6 in each group with AF induction) (figure 1*d*). Without treatment, SNRTs, AV node conduction function are indistinguishable between *Pak1* cKO and *Pak1*^fl/fl^ control groups (*n* = 3, *n* = 4 respectively, *p* > 0.05; figure 1*e–h*). Masson's trichrome showed that the overall collagen contents and fibrosis are hardly stained in both the *Pak1*^fl/fl^ control and *Pak1* cKO groups (figure 1*i*).

**Table 1.** Summarized surface ECG parameters between *Pak1* conditional knock out and control mice at four months old.

genotype		*n*	heart rate (BPM)	RR interval (ms)	P duration (ms)	PR interval (ms)	QRS duration (ms)	QT Interval (ms)	QTc (ms)
baseline	*Pak1^f^*^l/fl^	24	452.0 ± 7.0	133.7 ± 2.0	15.7 ± 0.5	33.6 ± 0.6	9.2 ± 0.4	13.6 ± 0.4	37.2 ± 1.1
	*Pak1* cKO	17	434.8 ± 12.2	140.0 ± 3.5	16.2 ± 0.9	34.3 ± 0.6	8.3 ± 0.4	13.7 ± 0.4	36.6 ± 1.2

### (b) *Pak1* deficiency alters atrial action potential, calcium transient and increases susceptibility to alternans *ex vivo*

**Figure 2.**
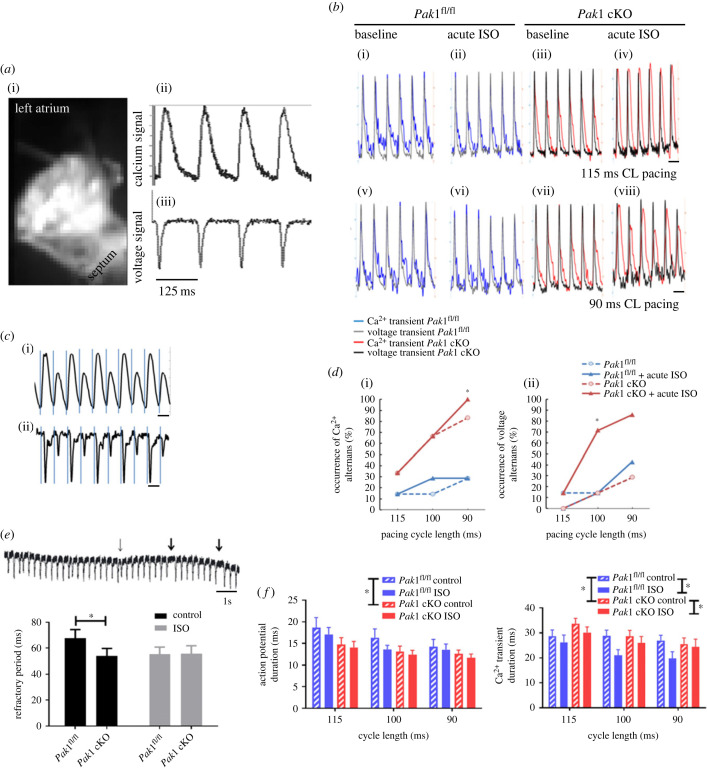
Optical mapping from atria of *Pak1* conditional knock out and *Pak1*^fl/fl^ control mice. (*a*) Representative example image of a left atrium dual loaded with calcium (Rhod-2) and voltage (RH-237) dyes and raw signals extracted from this image from the calcium (*a*ii) and voltage (*a*iii) channels. (*b*) Left atria (LA) representative recordings in *Pak1* conditional knock out (cKO) and *Pak1*^fl/fl^ mice during dynamic pacing at 90 and 115 ms cycle lengths (CL) in control and ISO challenge conditions. *Pak1*^fl/fl^ : blue trace–Ca^2+^ transients; grey trace–voltage signals; *Pak1* cKO: red trace–Ca^2+^ transients; black trace–voltage signals; the two signals recorded simultaneously in the same region of interest were superimposed by normalizing their maximum amplitudes to 100 and minimum to 0 (arbitrary units). (*c*) Representative examples of amplitude alternans in calcium (*e*i) and voltage (*e*ii) raw fluorescence signals from murine LA during dynamic pacing protocols. Alternans were defined as an average 15% change in signal amplitude between consecutive beats for at least six beats. (*d*) Line graphs to show cumulative occurrence of alternans in *Pak1*^fl/fl^ and *Pak1* cKO tissues under dynamic pacing, under both control and ISO (10 nM) conditions. Pak1 cKO LA are significantly more prone to the development of calcium transient (i) and action potential (ii) alternans upon ISO challenge. The asterisk indicates significant difference between genotypes in the presence of ISO (*p* < 0.05, Fisher's exact test). (*e*) The tendency to develop arrhythmogenic events in LA of *Pak1*^fl/fl^ and *Pak1* cKO mice. Refractory period (ms) of LA tissue from *Pak1*^fl/fl^ and *Pak1* cKO mice measured using S1S2 pacing protocol. Upon application of ISO (10 nM), a significant decrease in the refractory period from 65.7 ± 6.8 ms to 55.5 ± 5.3 ms was observed in the *Pak1*^fl/fl^ group (*p* < 0.05, two-way ANOVA with Sidak-corrected post-hoc testing). *Pak1* cKO-LA showed no significant difference in refractoriness between control and ISO challenge conditions (54.0 ± 5.8 ms to 55.8 ± 6.0 ms). Data are displayed as mean ± s.e.m.; *n* = 8 in *Pak1*^fl/fl^ group, and *n* = 9 in *Pak1* cKO group with the asterisk indicating *p* < 0.05. (*f*) Left panel: action potential duration (APD; as time to 75% recovery, in ms) is significantly shorter in LA from *Pak1* cKO across a range of pacing cycle lengths when compared to *Pak1*^fl/fl^ (*n* = 16 regions from eight atria for *Pak1* cKO and 18 regions from nine atria for *Pak1*^fl/fl^). The asterisk indicates difference between conditions as a whole (*p* < 0.05, multivariate ANOVA). No significant effect of ISO (10 nM) was observed on APD in Pak1 cKO. Right panel: calcium transient duration (CaTD; as time to 75% recovery, in ms) is significantly longer in LA from Pak1 cKO across a range of pacing cycle lengths when compared to *Pak1*^fl/fl^ (*n* = 15 regions from eight atria for both genotypes). Atria from both genotypes responded to ISO treatment (10 nM) with a significant reduction in CaTD. An asterisk indicates significant difference between conditions as a whole (*p* < 0.05, multivariate ANOVA).

Dual-dye mapping of left atria (LA) from *Pak1^fl/fl^* and *Pak1* cKO mouse hearts was carried out to further probe the properties of the atrial tissue which might underlie the arrhythmias seen *in vivo*. A representative example of dye-loaded tissue and raw signals are given in figure 2*a*i–iii.

During dynamic pacing, *Pak1* cKO LA tissue exhibited significant differences in the propensity to develop both Ca^2+^ and voltage amplitude alternans (representative examples of recordings in control and ISO conditions at 90 and 115 ms cycle lengths (CL) pacing in LA are shown in figure 2*b*). Ca^2+^ transients and APs at CL of 90, 100 and 115 ms were analysed for occurrence of arrhythmic events. The three time points were chosen in order to capture normal pacing in control conditions before any tissue develops a second degree of block (2 : 1 block). *Pak1* cKO-derived LA displayed a higher percentage of Ca^2+^ amplitude alternans than *Pak1^fl/fl^*. This tendency was maintained across control and ISO challenge conditions and statistically significant when comparing at 90 ms CL (*p* < 0.05, Fisher's exact test). Pak1 cKO atria were similar to *Pak1*^fl/fl^ in their propensity to develop AP amplitude alternans under control conditions but showed a significantly increased tendency in the presence of ISO, particularly at 100 ms CL (*p* < 0.05, Fisher's exact test) (figure 2*c,d*). While 100% and 85.71% of the *Pak1* cKO mice developed Ca^2+^ and voltage alternans respectively, only 28.57% and 42.86% of *Pak1*^fl/fl^ mice showed arrhythmogenic behaviour at 90 ms CL pacing in presence of acute β-adrenergic stimulation.

In order to determine the refractory period of the tissue (figure 2*e*), an S1S2 protocol was applied. The time corresponding to the first cycle at which the S2 failed to trigger an AP was used to calculate the refractory period (figure 2*e*, first thick arrow). The refractory period measured from control *Pak1^fl/fl^* atria was 63.7 ± 6.5 ms (*n* = 7). As predicted, ISO caused a significant decrease in refractory period in *Pak1^fl/fl^* atria, to 52.9 ± 5.3 ms (*n* = 7, *p* < 0.05, ANOVA with Sidak's *post-hoc* correction). The refractory period measured in *Pak1* cKO atria in the absence of ISO had a tendency to be shorter than that of *Pak1^fl/fl^*, at 49.0 ± 3.3 ms (*n* = 8) but did not change significantly on stimulation with 10 nM ISO (51.0 ± 4.1 ms, *n* = 8, *p* > 0.99 ANOVA with Sidak's *post-hoc* correction).

A dynamic pacing protocol was applied to each LA tissue under both control and ISO (10 nM) conditions to measure APD and calcium transient duration (CaTD) and assess the development of alternans. Analysis of APD at 75% recovery from the measured AP peak across the dynamic protocol, revealed significantly shorter APs in *Pak1* cKO than in *Pak1*^fl/fl^ derived LA under control conditions (*p* < 0.05, multivariate ANOVA; initial n*_Pak1_*_fl/fl_ = 16 regions from eight atria and n_Pak1 cKO_ = 18 regions from nine atria respectively). No significant change in APD was observed upon exposure to ISO when compared to baseline conditions (figure 2*f*). This is consistent with the lack of change in refractory period observed in *Pak1* cKO mice in the presence of ISO (figure 2*e*). With regard to CaTD, atria from *Pak1* cKO exhibited a significantly longer CaTD than their *Pak1*^fl/fl^ counterparts (figure 2*f*; *p* < 0.05, multivariate ANOVA; initial n*_Pak1_*_fl/fl_ = 16 regions from eight atria and n_Pak1 cKO_ = 18 regions from nine atria respectively). Additionally, the CaTD from both genotypes responded to ISO application via shortening of duration. To maintain consistency with the arrhythmia analysis, the APD and CaTD for the same 90, 100 and 115 ms pacing intervals are plotted in figure 2*f*. For instance, at 115 ms CL pacing during control conditions APD was 18.39 ± 2.4 ms in *Pak1*^fl/fl^ and 14.74 ± 1.6 ms in *Pak1* cKO (115 ms group interval).

### (c) *Pak1* deficiency alters RyR2 open probability characteristics

**Figure 3.**
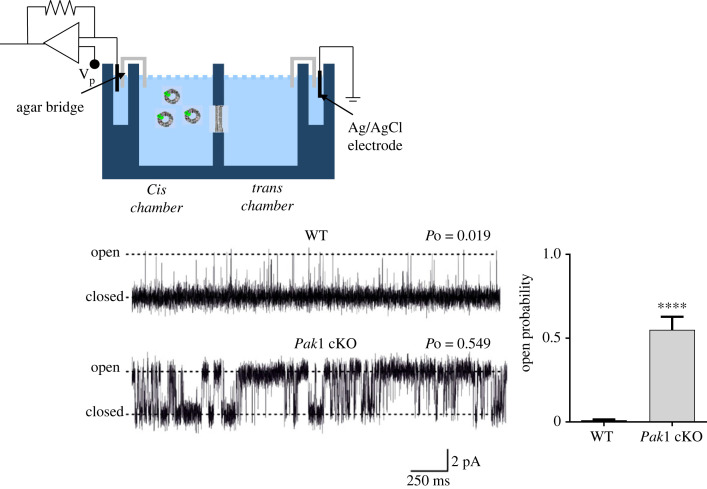
RyR2 single channel recording from atria of *Pak1* conditional knock out and wild-type (WT) control mice. Representative single-channel recordings of RyR2 from WT (top) and *Pak1* conditional knock out (cKO, bottom) mice under control conditions (10 µM cytosolic Ca^2+^). The bar-charts compares the mean open probability values for the RyR2 channels from the WT (*n* = 7) and *Pak1* KO (*n* = 10) mice (s.e.m., ****: *p* < 0.0001).

Figure 3 compares representative RyR2 single-channel recordings obtained from WT and *Pak1* cKO hearts in presence of 2 µM cytosolic Ca^2+^. Mean Po was significantly higher for channels derived from *Pak1* cKO hearts (0.550 ± 0.078 s.e.m.; *n* = 10) compared to those obtained from WT control hearts (0.010 ± 0.005 s.e.m.; *n* = 7; *p* < 0.0001) (figure 3).

### (d) *Pak1* deficiency acts through modifying the phosphorylation of CaMKII*δ* and RyR2, as well as expression of *Serca2a* and *Ncx* at transcriptional level

**Figure 4.**
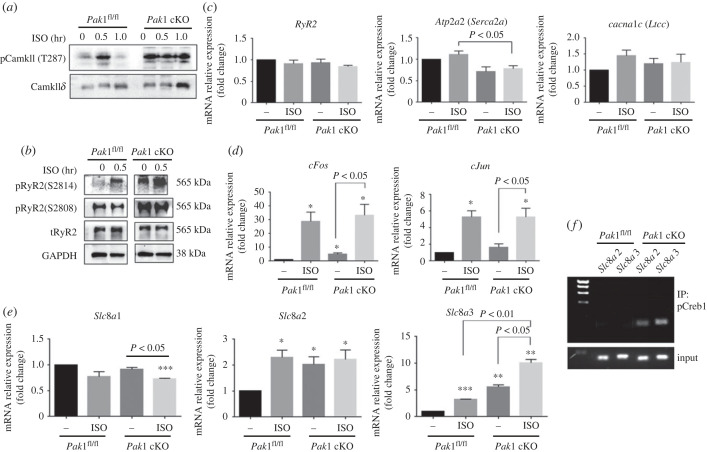
Activity of the CamkII-mediated RyR2 phosphorylation and AP-1*/*pCREB1*/Slc8a* signalling in atria of *Pak1* conditional knock out (*n* = 3) and *Pak1*^fl/fl^ control mice (*n* = 3). (*a*,*b*) Phosphorylation of CamkII*δ* (T287) (*a*) and RyR2 (S2814 and S2808) (*b*) was investigated in left atria of *Pak1*^fl/fl^ and *Pak1* cKO groups following intraperitoneal injection (i.p.) injection of ISO (10 mg kg^−1^). (*c–e*) Expression of *RyR2*, *Atp2a2 (Serca2a)*, *Cacna1c (Ltcc)* (*c*), AP1 components including *cFos*, *cJun* (*d*) as well as *Slc8a*1-3 (*Ncx*1-3) (*e*) genes was evaluated in atria of *Pak1*^fl/fl^ and *Pak1* cKO mice after 1 h following i.p. injection of ISO. (*f*) Chromatin immunoprecipitation (ChIP) assay was carried out on the promoter region of *Slc8a2* and *Slc8a3*. The antibody of phosphor-CREB1 was used to investigate the interaction between pCREB1 and the promoter of *Slc8a2* and *Slc8a3*. Statistical analysis was performed with a Student *t*-test. Values are mean ± s.e.m., *p < 0.05, **p < 0.01, ***p < 0.001 versus *Pak1*^fl/fl^ mice group in figure 4*c–e.*

We explored the possible molecular mechanisms underlying atrial arrhythmogenesis by investigating the activity and expression of key Ca^2+^ handling proteins at the protein or transcript levels. In LA from *Pak1*^fl/fl^ control mice, CamkII*δ* (T287) and RyR2 (S2814) were phosphorylated at 0.5 h after treatment with ISO. LA from *Pak1* cKO manifested a dramatically raised phosphorylation of CamkII*δ* (T287) in without stimulation, which was maintained after exposure to ISO (figure 4*a*). *Pak1* deficiency in the atria leads to the hyper-phosphorylation of RyR2 at two phosphorylation sites, S2808 and S2814, both at rest and in response to ISO (figure 4*b*). Such a hyper-phosphorylated state may give rise to abnormal heart beating owing to breaking Ca^2+^ homeostasis in the atria. Hence, the transcription level of Ca^2+^ homeostasis regulators including *RyR2*, *Atp2a2 (Serca2a)* and *Cacna1c (Ltcc)* were detected in atria of *Pak1* cKO mouse hearts in the absence or presence of ISO. The results showed that *Serca2a* mRNA expression was significantly reduced in *Pak1* cKO mice following ISO treatment compared with the *Pak1*^fl/fl^ mice (figure 4*c*).

Myocardial intracellular increased Ca^2+^ evokes the CREB1 pathway, which is activated by CaMKII. CaMKII*δ* and CREB1 transcriptionally regulate target genes which are composed of cFOS, cJUN and CREB1. The cFOS and cJun genes are very famous as proto-oncogenes, are immediate early genes in cardiomyocytes, and associated with hypertrophy [28–32]. We wondered whether provoked phosphorylation of CamkII*δ* (T287) in *Pak1* cKO influences the transcriptional activation of *cFos* and *cJun* by the activation of Creb1 in the absence or presence of ISO. As shown in figure 4*d*, atria of *Pak1* cKO mice showed a significantly elevated transcription level of AP1 components including *cFos* and *cJun* when compared to *Pak1*^fl/fl^ mice in the absence of ISO. ISO activated transcription levels of these genes remarkably in both the *Pak1*^fl/fl^ and *Pak1* cKO atria. We did not observe a difference in the expression level of AP1 components including *cFos* and *cJun* between *Pak1*^fl/fl^ mice and *Pak1* cKO mice after ISO stimulation (figure 4*d*), which means these genes are more triggered by the β-adrenergic stress than *Pak1* in atria and there is less possibility that *cFos* and *cJun* are the direct target of the phosphor-CamkII*δ* (T287)/Creb1 influenced by *Pak1*.

Given the conclusions of DeSantiago *et al*. [18] regarding PAK1 activation as a protection against NCX-induced arrhythmias, we tested the transcriptional activation of *Slc8a* (*Ncx*) isotypes in *Pak1*^fl/fl^ and *Pak1* cKO mouse atria in the presence and absence of ISO to investigate whether the CamkII*δ*/Creb1 pathway induces *Slc8a* expression. As shown in figure 4*e*, in the absence of ISO stimulation, atria of *Pak1* cKO mice were shown to exhibit a significantly increased expression level of Slc8a2 and Slc8a3 (by twofold and fivefold respectively) compared to *Pak1*^fl/fl^. ISO treatment increased the expression of *Slc8a3* in atria from both genotypes by more than twofold when compared to their level in the absence of stimulation, maintaining the significant difference between *Pak1*^fl/fl^ and *Pak1* cKO. This rendered the expression level of *Slc8a3* in ISO-stimulated *Pak1* cKO 10-fold intensified from the non-stimulated *Pak1*^fl/fl^. Atria from *Pak1*^fl/fl^ mice responded to ISO stimulation by significantly increasing (by more than twofold) expression of *Slc8a2*, whilst in *Pak1* cKO ISO induced little further change. The expression level of *Slc8a2* was therefore preserved in the *Pak1* cKO mice at the same level as *Pak1*^fl/fl^ mice injected with ISO. *Slc8a1* expression, conversely, was rather slightly, but significantly decreased in *Pak1* cKO mice atria stimulated by ISO (figure 4*e*). These data indicate that the constitutive increased phosphorylation of CaMKII*δ* in *Pak1* deficient atria induced the transcriptional expression *Slc8a2* and *Slc8a3*. Consistently increased expression of *Slc8a* could lead to a defect in systemic Ca^2+^ flux through *Slc8a* and may lead to the production of delayed afterdepolarizations developing arrhythmia in *Pak1* deficient mice.

The next question was what makes *Slc8a2* and *Slc8a3* transcription increase? We considered the possibility of CREB1 interacting with the promoter region of *Slc8a2* and *Slc8a3* isotypes. Atria were isolated from *Pak1*^fl/fl^ and *Pak1* cKO and the question of whether pCreb1 binds to the promoter regions of *Slc8a2* and *Slc8a3* was explored through ChIP assay. As shown in figure 4*f*, pCreb1 was found to be interacting with both of *Scl8a2* and *Slc8a3* promoters in *Pak1* cKO mice, which means that phosphorylation of CamkII*δ* observed in *Pak1* cKO mice atria induced phosphorylation of Creb1, which led to an increase in transcription of *Slc8a2* and *Slc8a3*.

## 4. Discussion

Our whole animal, tissue, cellular and molecular studies together demonstrate for the first time, to our knowledge, key regulatory roles for *Pak1* in cellular adaptation to acute adrenergic stress, through regulatory actions on the activity of RyR2 and CamkII and expression of *Serca2a* and *Ncx* genes accordingly critical to Ca^2+^ homeostasis and electrophysiological stability in the atria. These probably involve both post-translational and transcriptional mechanisms. Our studies provide new insights into the Ca^2+^ homeostasis regulatory mechanism in the atria and translational platform for designing novel Pak1-based therapeutic strategies for atrial tachyarrhythmias.

Our electrophysiological studies in intact animals and isolated atrial independently implicated *Pak1* in maintaining atrial electrophysiological stability and Ca^2+^ homeostasis during both baseline and acute adrenergic stress through acting on triggered activity rather than re-entry. Concurring with findings in the atria from total *Pak1* knockout mice [18] that showed correspondingly occurrence of frequent, spontaneous premature atrial contractions at tissue level, and more pronounced increase in diastolic [Ca^2+^]_i_ under Ang II stress at single cell level.

Ca^2+^ alternans recorded in LA in particular when they are acutely exposed to isoprenaline stress in our study suggests the alteration of Ca^2+^ homeostasis that could be linked to either abnormal SR Ca^2+^ release or decrease in kinetics of SERCA2A and NCX currents and an increase in diastolic Ca^2+^ levels in as we found in Pak1 cKO mouse ventricular cardiomyocytes under baseline conditions [14]. Upon isoprenaline treatment it is expected that the described changes become more pronounced. A blockade of Ca^2+^ reuptake into the SR via SERCA2A inhibition using the antagonist thapsigargin was demonstrated to increase the susceptibility to cellular Ca^2+^ alternans [33]. Additionally, Cutler *et al*. [34] have shown that increasing the SERCA2A expression significantly lowers the occurrence of cytosolic calcium alternans. With regard to the observed increase in voltage alternans in the isoprenaline-exposed cardiac specific knockout mice compared to WT counterparts, one of the two hypotheses describing the mechanisms of alternans generation suggested that AP alternans may appear as a secondary effect owing to an alteration in calcium-sensitive electrogenic sarcolemmal ionic currents during cytosolic calcium alternans [34].

We previously [14] have also observed an increase in SR Ca^2+^ refilling time in Pak1 cKO ventricular myocytes when compared to WT littermates. This observation along with slow NCX and SERCA2A kinetics, may imply longer cytosolic Ca^2+^ transients. In fact, ventricular myocyte experimentation using total *Pak1* KO mice have reported longer Ca^2+^ transient recovery time in KO than in WT mice [35]. The two studies, although carried out on ventricular myocytes, are in line with the longer CaTDs identified in the LA of *Pak1 cKO* mice under baseline conditions in the present study.

Our data on RyR2 single-channel recordings revealed that channels derived from PAK1 cKO hearts exhibit a markedly higher Po than channels from control hearts, suggesting an increase in cytosolic Ca^2+^ sensitivity of RyR2 channels from PAK1 cKO mice. This increase in RyR2 activity might have resulted from the higher phosphorylation levels at S2808 and S2814 detected in the PAK1 cKO heart preparations since enhanced phosphorylation at these sites has been linked to RyR2 dysregulation and increased RyR2-mediated cytosolic Ca^2+^ leak [36–39]. The altered RyR2 responses to Ca^2+^ would probably impact on the consistency of local SR Ca^2+^-release events, providing an explanation of the cellular basis for an increase in arrhythmogenesis and abnormal Ca^2+^ transient observed in Pak1 cKO mice.

Our molecular studies then associated PAK deficiency in *Pak1* cKO mice with alterations of the activity of RyR2 and CamKII and expression of Serca2a and Ncx genes that are essential to Ca^2+^ homeostasis and electrophysiological stability in the atria. These changes may reflect multiple regulatory mechanisms that PAK1 is involved in Ca^2+^ handling proteins activity and expression at both post-translational and transcriptional levels. Firstly, in our previous study, we have established the action of PAK1 on the phosphatase PP2A and the resulting balance between kinase and phosphatase activity controlling LTCC and I_K_ activity in sinoatrial node pacemaker cells [7]; An important difference between atrial and ventricular muscle concerns the resting activity of adenylyl cyclase: in atrial myocytes there is significant turnover in cAMP-dependent pathways even in the absence of β-adrenocpetor stimulation [17]. As a consequence, it is predicted that the roles of phosphatase (PP2A) activation by Pak1 will be enhanced in atrial tissue. Previous studies have established reciprocal action of protein kinases CaMKII and PKA on RyR2 phosphorylation sites S2031, S2808 and S2814 are opposed by protein phosphatases PP1, PP2A and PP2B [40]. Thus Pak1 deficiency resulting in balance between PKA, CamkII and PP2A is likely to underlie the enhanced RyR2 phosphorylation observed in *Pak1* cKO mouse atrial tissue. Admittedly, such mechanisms require more investigations in the future, while the downregulated *SERCA2a* and upregulated Slc8a2 and Slc8a3 transcripts in Pak1 cKO atrial tissue may reflect a complicated transcriptional regulation but important role of Pak1 as a regulator of cardiac SERCA2a and Slc8a2 and Slc8a3 through transcriptional mechanisms, which is consistent with our previous finding in the ventricular tissue [14].

In conclusion, our study demonstrates for the first time, to our knowledge, a key regulatory role of Pak1 for maintaining electrophysiological stability and Ca^2+^ homestasis in the atria through regulation of calcium handling proteins RyR2, Serca2a and NCX via either post-translational and transcriptional mechanisms, thus providing new insights into atrial Ca^2+^ signalling regulatory mechanism that has an implication in developing new therapeutic strategies for atrial tachyarrhythmias.

## Data Availability

The data are provided in the electronic supplementary material [41].
