# Correction to: ‘Cardiac deficiency of P21-activated kinase 1 promotes atrial arrhythmogenesis in mice following adrenergic challenge’

**DOI:** 10.1098/rstb.2023.0143

**Published:** 2023-07-17

**Authors:** Eunjeong Jung, Rebecca Capel, Congshan Jiang, Elisa Venturi, Georgiana Neagu, Sarah Pearcey, Yafei Zhou, Yanmin Zhang, Ming Lei

*Phil. Trans. R. Soc. B* **378**, 20220168. (Published online 1 May 2023) (https://doi.org/10.1098/rstb.2022.0168)

Errors in table 1 were identified after publication—the table column headings were incorrectly associated with the data. The data themselves are unchanged.

The corrected table is shown below and has also been corrected in the article.

**Table 1.** Summarized surface ECG parameters between *Pak1* conditional knock out and control mice at four months old.

	genotype	*n*	heart rate (BPM)	RR interval (ms)	*P* duration (ms)	PR interval (ms)	QRS duration (ms)	QT interval (ms)	QTc (ms)
baseline	*Pak1^f^* ^l/fl^	24	452.0 ± 7.0	133.7 ± 2.0	15.7 ± 0.5	33.6 ± 0.6	9.2 ± 0.4	13.6 ± 0.4	37.2 ± 1.1
	*Pak1* cKO	17	434.8 ± 12.2	140.0 ± 3.5	16.2 ± 0.9	34.3 ± 0.6	8.3 ± 0.4	13.7 ± 0.4	36.6 ± 1.2

